# Vocal specialization through tracheal elongation in an extinct Miocene pheasant from China

**DOI:** 10.1038/s41598-018-26178-x

**Published:** 2018-05-25

**Authors:** Zhiheng Li, Julia A. Clarke, Chad M. Eliason, Thomas A. Stidham, Tao Deng, Zhonghe Zhou

**Affiliations:** 1 Key Laboratory of Vertebrate Evolution and Human Origins, Institute of Vertebrate Paleontology and Paleoanthropology, Chinese Academy of Sciences, Beijing, 100044 China; 2 CAS Center for Excellence in Life and Paleoenvironment, Beijing, 100044 China; 3 Department of Geological Sciences, University of Texas at Austin, Austin, Texas 78712 United States; 4 Integrative Research Center, Field Museum of Natural History, Chicago, Illinois 60605 United States

## Abstract

Modifications to the upper vocal tract involving hyper-elongated tracheae have evolved many times within crown birds, and their evolution has been linked to a ‘size exaggeration’ hypothesis in acoustic signaling and communication, whereby smaller-sized birds can produce louder sounds. A fossil skeleton of a new extinct species of wildfowl (Galliformes: Phasianidae) from the late Miocene of China, preserves an elongated, coiled trachea that represents the oldest fossil record of this vocal modification in birds and the first documentation of its evolution within pheasants. The phylogenetic position of this species within Phasianidae has not been fully resolved, but appears to document a separate independent origination of this vocal modification within Galliformes. The fossil preserves a coiled section of the trachea and other remains supporting a tracheal length longer than the bird’s body. This extinct species likely produced vocalizations with a lower fundamental frequency and reduced harmonics compared to similarly-sized pheasants. The independent evolution of this vocal feature in galliforms living in both open and closed habitats does not appear to be correlated with other factors of biology or its open savanna-like habitat. Features present in the fossil that are typically associated with sexual dimorphism suggest that sexual selection may have resulted in the evolution of both the morphology and vocalization mechanism in this extinct species.

## Introduction

A key characteristic of nearly all living birds is their ability to vocalize and produce a variety of sounds for acoustic signaling and communication. The majority of these sounds are produced by the avian vocal organ, or syrinx, located at the tracheobronchial junction. The syrinx likely was present in the ancestral crown group bird and evolved by the end of the Cretaceous^1^. However, fossil syrinxes are rare^1,2^ compared to more commonly preserved cartilaginous and ossified rings of the trachea in crown birds^1,3^. This near absence of fossil evidence has made the reconstruction of the evolution of vocal behavior in extinct birds difficult, although phylogenetic and molecular evidence has offered clues^4–6^.

Sounds produced by the syrinx may be modified by the upper vocal tract, including the shape of the oropharyngeal cavity affected by the mouth and tongue^6,7^, tracheal bullae^8,9^ and modifications to the diameter of the trachea, expansion of the esophagus while vocalizing^5,7,10^, and significant tracheal elongation (TE) where the trachea forms permanent coils or loops^11,12^. Significant elongation of the trachea has likely evolved many times in birds^5^ and is known to occur in at least 60 species, including species of the extinct giant moas^12^, Galliformes (Cracidae, Numinidae, and Tetraoninae), Gruiformes (Gruidae), Ciconiiformes (Threskiornithidae), Charadriiformes (Rostratulidae), and songbirds^11^. There are a number of different ways that TE occurs in birds including the trachea extending into a single loop or multiple loops subcutaneously over the pectoral muscles caudally (even to the caudal region of the body), the lengthened trachea overlaying only the lateral surface of the pectoral muscles, extension of the trachea into the body of the sternum or furcula itself, or looping of the trachea intrathoracically^13–15^.

Many hypotheses have been proposed for the evolution and function of TE in birds including production of lower fundamental frequencies or increased amplitudes (for increased signal propagation in particular habitats), and size exaggeration (i.e., sounding like a larger bodied bird), either to attract a potential mate or startle a predator, and even temperature or CO_2_ regulation, water retention, and heat conservation^11^. Among these hypotheses, thermoregulation and vocalization have been thought to be the most likely factors that result in tracheal elongation^16^. Positive selection for a longer trachea may occur in birds to compensate for limited nasal turbinate sizes, aiding in the reduction of evaporative heat and water loss^16^. However, this hypothesis does not appear to explain its absence in birds of similar ecologies to those exhibiting TE or dimorphism in the trait within a single species. Size exaggeration is another hypothesis for the evolution of TE in many clades of birds that can explain cases of sexual dimorphism in TE^11,17^. An elongated trachea allows birds to produce a vocalization similar to that of a larger-bodied bird by manipulating the acoustic output^11^. Size selection sometimes acts mainly on male individuals and female preferences for low-frequency sounds might explain why males tend to have longer tracheas than females in some clades. The role of TE in vocal tract filtering remains unclear^10,11,18^, but the filtering is known to occur frequently and be more important than previously thought in bird vocalziation^19^.

Here, we describe a new late Miocene (~7–11 Ma) fossil of a pheasant from Gansu Province, northwest China that preserves the oldest known instance of TE in a bird, as part of a rare nearly complete skeleton. The new species is placed phylogenetically within a polytomy including *Perdix*, a few members of Phasianinae, Tetraoninae, and Meleagridinae (Galliformes: Phasianidae). TE is present in many extant galliform birds, including most male cracids (e.g., *Ortalis veula*), male *Tetrao urogallus* (Tetraoninae), and both sexes of *Guttera*^11,13^ (Numididae). In addition to Tetraoninae, this taxon represents the third record of elongation of the trachea in Phasianidae (besides *Tetrao urogallus* and *Lagopus mutus*) and the first within Phasianinae, a diverse group including over 150 extant species of pheasants, peafowl, francolins, and others. In addition, this new species from the Miocene deposits of Gansu Province adds new data to the ecological diversity of this extinct steppe-dwelling avifauna at the edge of the Tibetan Plateau that also includes an extinct long-legged kestrel, ostrich, and Old World vultures^20–23^.

## Results

### Systematic paleontology

AVES Linnaeus, 1758

GALLIFORMES Linnaeus, 1758

PHASIANIDAE Vigors, 1825

*Panraogallus hezhengensis* gen et sp. nov.

#### Etymology

The genus name is the pinyin of the Chinese characters meaning ‘coiling’ and the Latin for ‘chicken,’ referring to the preserved elongate trachea in this species. The specific epithet, ‘hezhengensis’ refers to Hezheng area in the Linxia Basin of Gansu Province where abundant fossils, including the holotype, have been collected.

#### Specimen number

HMV 1876 (HMV, Hezheng Paleozoological Museum, Vertebrate Collection, Gansu Province, China).

#### Locality and age

Baihua area near Zhuangkeji Township, Guanghe County, Gansu Province, China exposes the Liushu Formation that is late Miocene (7.25–11.1 Ma)^24^.

#### Holotype

**Figure 1:**
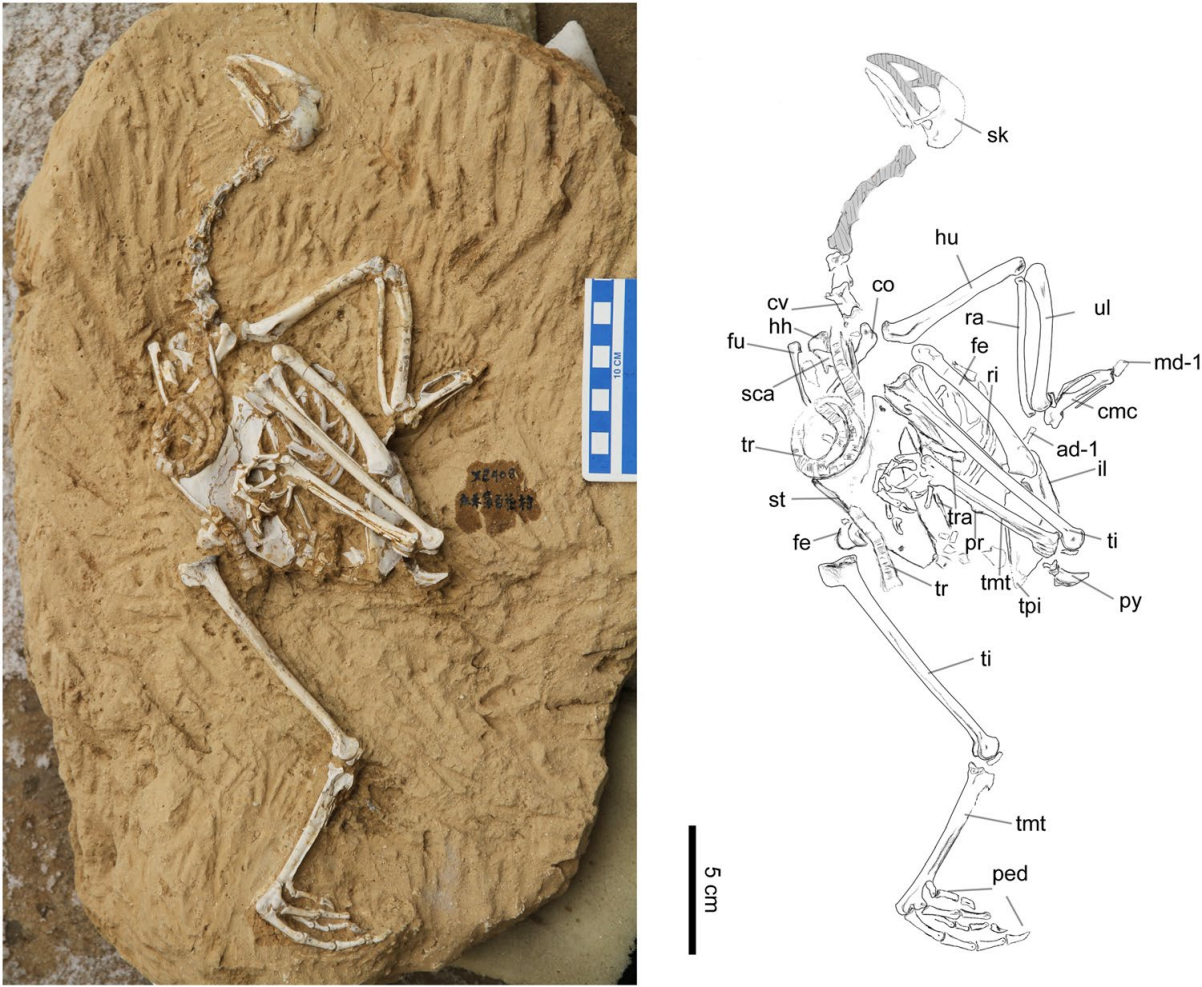
Photo and line drawing of the holotype specimen (HMV 1876). Anatomical abbreviations: co–coracoid, cmc–carpometacarpus, cv–cervical vertebrate, ad-1, first phalanx of alular digit; md-1–first phalanx of major digit, fe–femur, fu–furcula, hh–humeral head, hu–humerus, il–ilium, ped–pedal digits, pr–projection for the spur attachment, py–pygostyle, ra–radius, ri–rib, sca–scapula, sk–skull, st–sternum, ti–tibiotarsus, tmt–tarsometatarsus, tpi–terminal process of the ischium, tr–tracheal rings, tra–trabecula, and ul–ulna.

**Figure 2:**
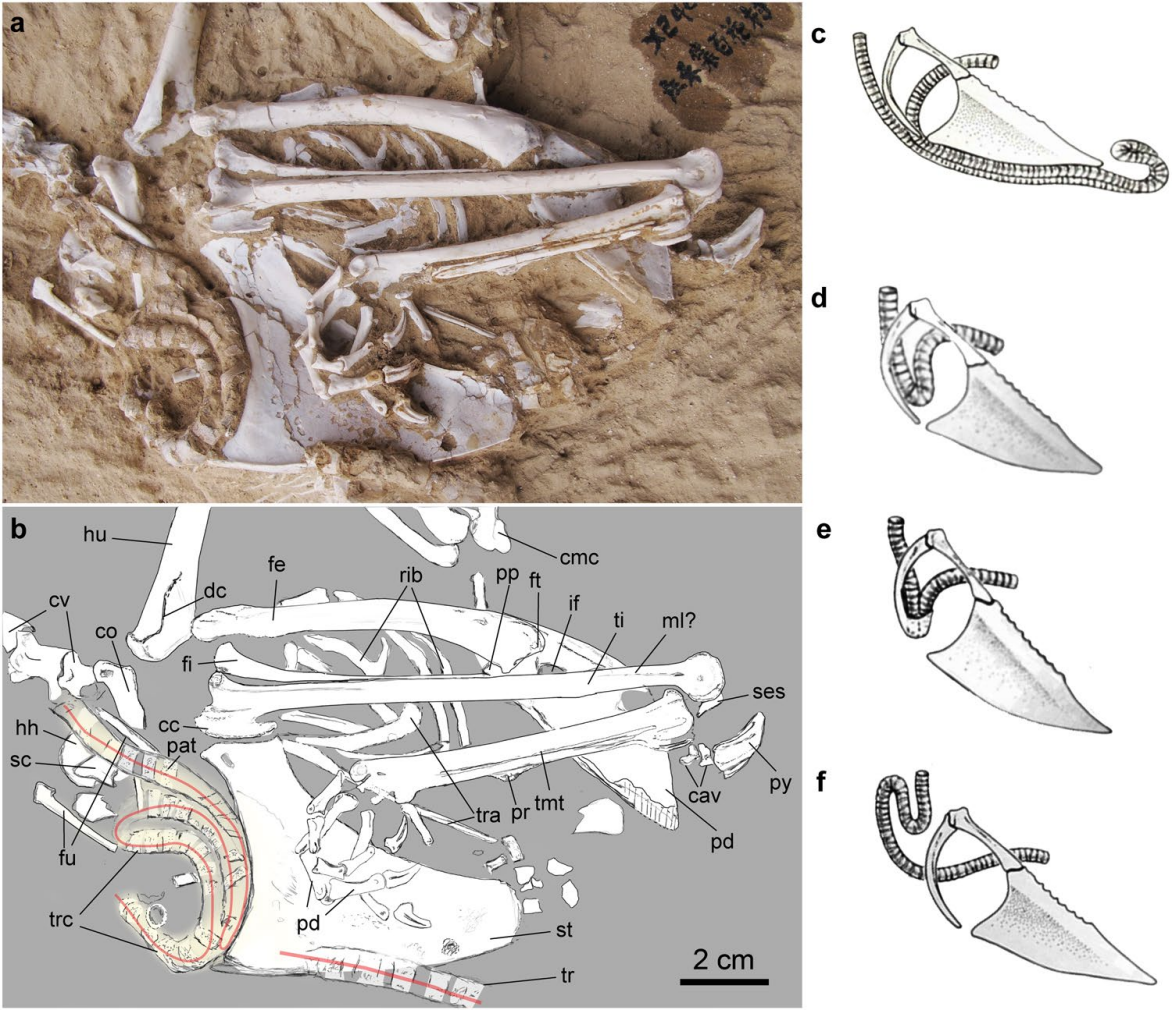
Photo and line drawing of the thoracic region of the holotype specimen of *Panraogallus hezhengensis* (HMV 1876). The tracheal loop is indicated by the red line and compared with that in the Helmeted curassow (*Pauxi pauxi*) (**c**), Black curassow (*Crax alector*) (**d**), Crested guineafowl (*Guttera pucherani*) (**e**), and Wood grouse (*Tetrao urogallus*) (**f**), which are adapted from ref.^48^. Anatomical abbreviations: cav–free caudal vertebrae, cc–cnemial crest, cmc–carpometacarpus, dc–deltopectoral crest, fi–fibula, ft–femur trochanteric crest, fu–furcula, hh–humeral head, if–ilioischiadicum foramen, ml?–muscular line, pat–patches, pd–pedal digits, pp–preacetabular process, py–pygostyle, pr–projection for the spur attachment on the tarsometatarsus, ses–sesamoid bone, st–sternum, tr–tracheal ring, tra–trabecula, and trc–tracheal coiling/loop.

**Figure 3:**
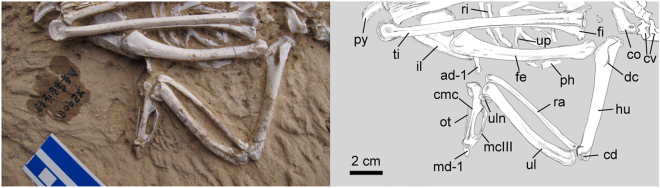
Photo and line drawing of the forearm and hind limb of the holotype specimen of *Panraogallus hezhengensis* (HMV 1876). Anatomical abbreviations: ad-1–first phalanx of the alular digit, co–coracoid, cmc–carpometacarpus, cv–cervical vertebrate, dc–deltopectoral crest, fe–femur, hu–humerus, il–ilium, mc iii–minor metacarpal, ot–ossified tendon, ph–phalanx, py–pygostyle, ra–radius, ri–rib, ti–tibiotarsus, tmt–tarsometatarsus, and ul–ulna.

**Figure 4:**
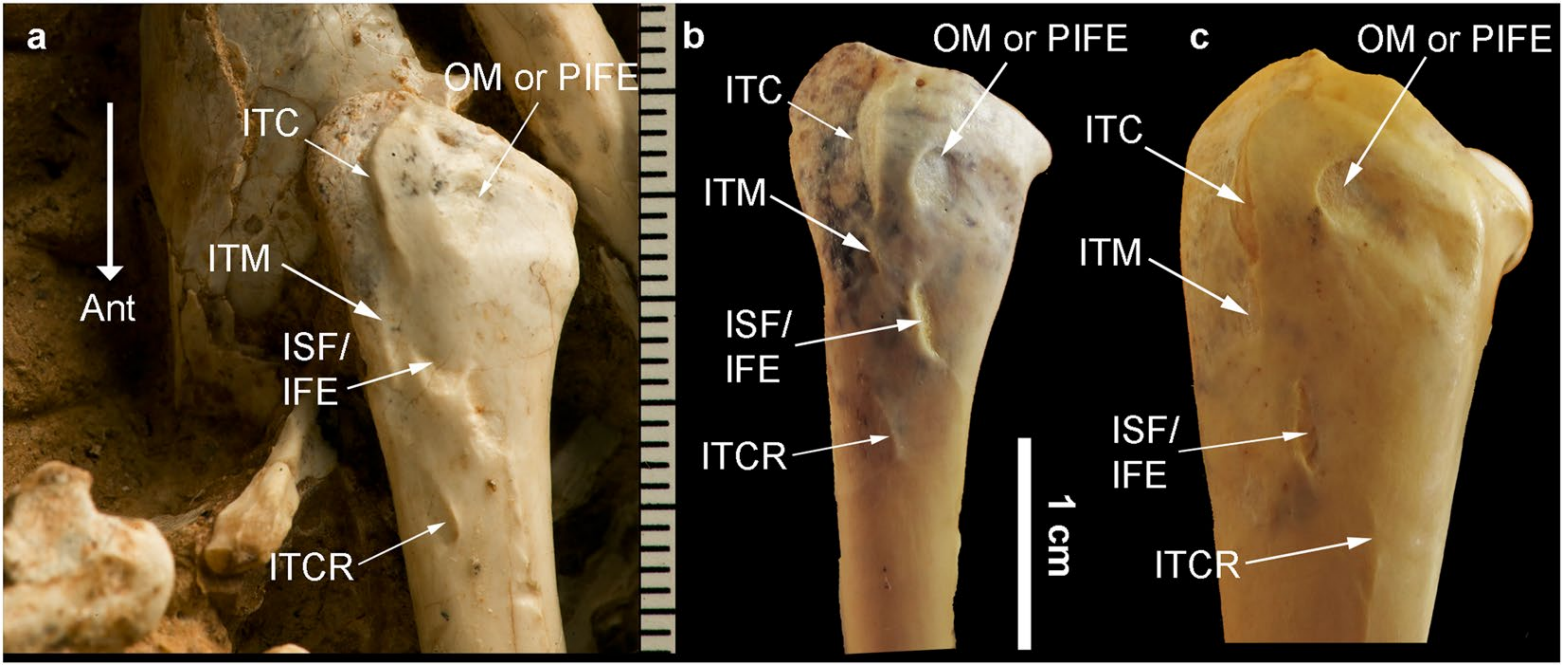
Photos of the left proximal femur in *Panraogallus hezhengensis* holotype (HMV 1876): (**a**) compared to that of *Tympanuchus phasianellus*, National Museum of Natural History, Smithsonian Institution, USNM 559940 (**b**), and *Lophophorus impejanus* USNM 347359 (**c**) in lateral view. Identification of hind limb muscular scars adopted from ref.^63^. Anatomical abbreviations: ISF/ IFE (m. iliofemoralis externus, m. ischiofemoralis), ITC (m. iliotrochantericus caudalis), ITCR (m. iliotrochantericus cranialis), and ITM (m. iliotrochantericus medius), OM or PIFE (obturatorius muscle or m. puboischiofemoralis). The white straight arrow (Ant) indicates the cranial direction.

**Figure 5:**
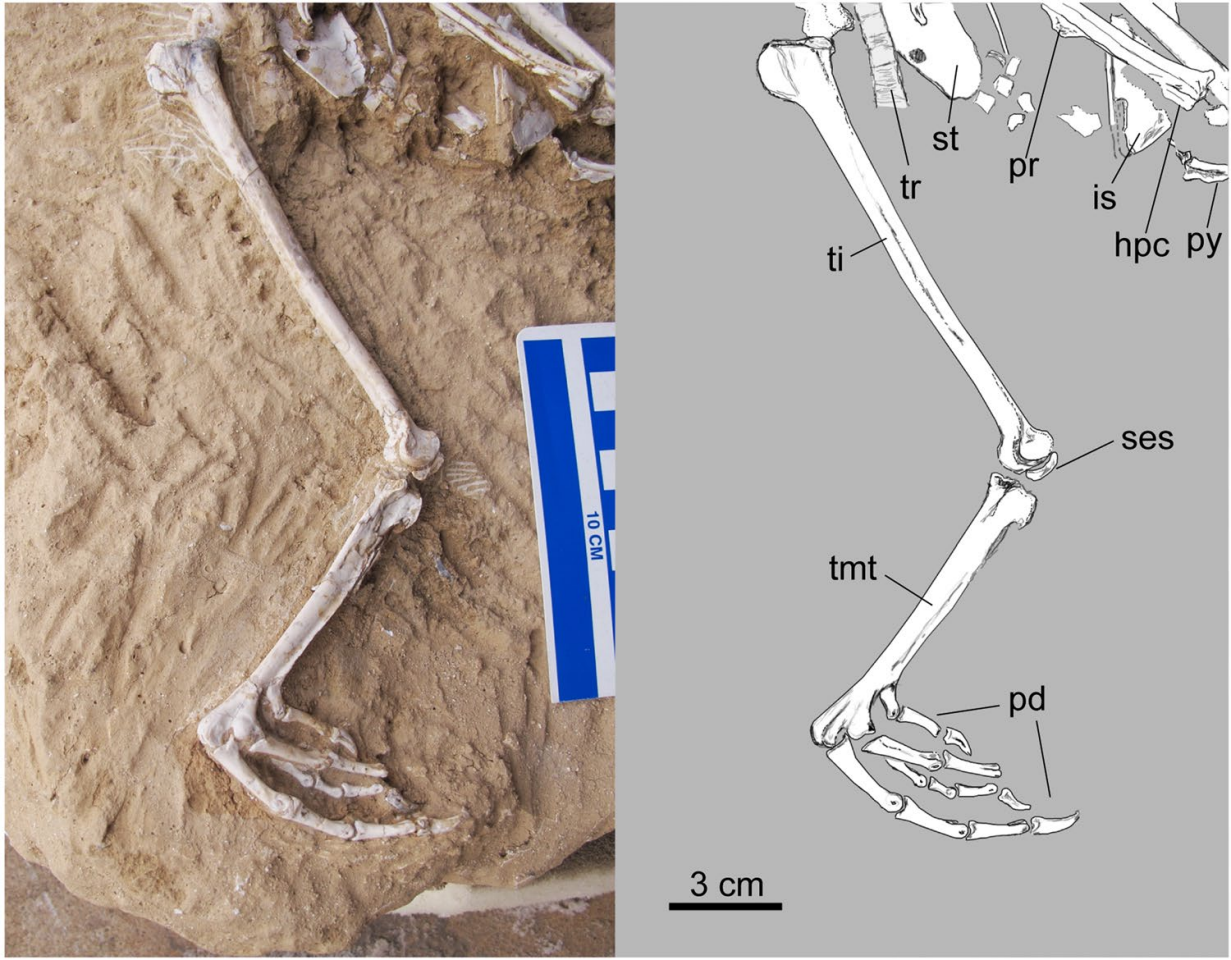
Left hind limb of the holotype of *Panraogallus hezhengensis* (HMV 1876). Anatomical abbreviations: hpc–hypotarsal crest, is–ischium, pd–pedal digits, pr–projection for the spur attachment on the tarsometatarsus, py–pygostyle, ses–sesamoid bone, st–sternum, ti–tibiotarsus, tmt–tarsometatarsus, and tr–tracheal rings.

**Figure 6:**
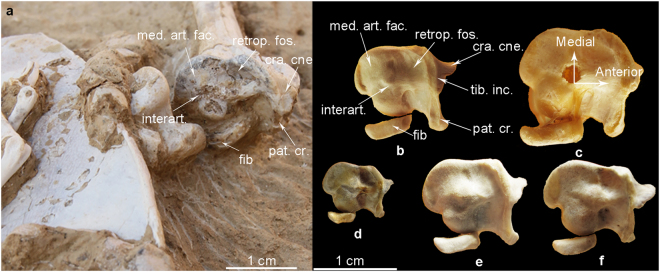
Photos of the proximal view of the left tibiotarsus in *Panraogallus hezhengensis* (HMV 1876) (**a**); with comparisons to various phasianids, *Tragopan temminckii* USNM 621027 (**b**), *Lophophorus impejanus* USNM 347359 (**c**), *Bonasa bonasia* USNM 586135 (**d**), *Dendragapus obscurus* USNM 502269 (**e**), and *Tympanuchus phasianellus* USNM 559940 (**f**). Anatomical abbreviations: cra. cne.–cranial cnemial crest, fib–fibula, interart.–interarticular area, med. art. fac.–medial articular facies, pat.cr.–patellar crest, retrop. fos.–retropatellar fossa, and tib. inc–tibia incisure.

The holotype specimen of *Panraogallus hezhengensis* is a nearly complete skeleton (Figs 1–6). It is from a young adult individual as demonstrated by the fusion of all major skeletal elements, a smooth periosteal surface on the bones, and formation of a tarsometatarsal process for spur attachment. Part of the skull, including the premaxillae, maxillae, nasofrontal region, and cranial cervical vertebrae were conspicuously reconstructed in plaster and other materials (grey regions, Fig. 1) prior to acquisition by the HMV; these parts are ignored in the description of the new taxon. The fossil preserves the jaw and caudal portion of the skull. The rest of the postcranial skeleton caudal to the penultimate caudal cervical vertebrae is unmodified original fossil material. The fossil is embedded in reddish clay, identical to that of other avian fossil specimens collected in the Linxia Basin in units attributed to the Liushu Formation^23^. The occurrence of TE and a bony blunt process for the attachment of a spur on the tarsometatarsus indicate that this individual may have been male (see below for more discussion).

#### Diagnosis

The new specimen differs from Cracidae and Megapodiidae in having a shorter and more robust femur^25,26^ and lacking an enlarged laterally-directed ridge on the lateral crest of the hypotarsus (for the passage of the m. fibularis longus tendon). *Panraogallus hezhengensis* also differs from Cracidae in having a strongly proximally-projected cranial cnemial crest^27,28^. In comparison to Megapodiidae, the Chinese specimen lacks a hooked acromion on the scapula and widely-splayed trochleae on the distal tarsometatarsi^25,29^. The presence of an enlarged intermetacarpal process is a derived feature present in both new world quail (Odontophoridae) and pheasants (Phasianidae)^30^. The intermetacarpal process of *Panraogallus* is similar to *Tragopan* and other core phasianids, but much larger than that of Odontophoridae^25^. The absence of a foramen on the caudal mandible additionally distinguishes the new specimen from other phasianids (e.g., turkeys [*Meleagris*], and grouse [Tetraoninae])^25,31^.

The new species is the third phasianid in addition to the Western Capercaillie (*Tetrao urogallus*) and Rock Ptarmigan (*Lagopus mutus*) that exhibits tracheal elongation: TE is a proposed autapomorphy of each species^32^. No extant species of Phasianinae has an elongate trachea, diagnosing the fossil as distinct from any living member of that clade. *Panraogallus* differs significantly from other phasianines (i.e. partridges, francolins, and Old World quails) in its sternal shape (with very deep caudal incisures), and in its significantly larger body size (estimated 2.5 kg). The size of the new specimen differentiates it from other Asian Neogene taxa. It is about twice the size of *Shandongornis*, which also differs in the absence of spur attachment on the tarsometatarsus, and is much larger than most other known extinct Asian Neogene galliform taxa^33–36^. Furthermore, this new species is smaller than extant species of pavonine pheasants (e.g., *Pavo* and *Argusianus)*, and other large-bodied phasianines. The species has a femur longer than the humerus in contrast to the state present in Cracidae, Numididae, Odontophoridae, and Tetraoninae^25^. In comparison with extinct members of Phasianidae, *Palaeocryptonyx* exhibits a second (dorsal) pneumotricipital fossa on the proximal humerus that is absent in the new species^37^; *Palaeortyx* is much smaller in size and has relatively shorter tarsometatarsus than *Panraogallus*^38^. The new species also differs from *Miogallus* in the morphology of the acrocoracoid process of the coracoid, which is more medially recurved in the new species^34–36^. Compared to *Tologuica*, the new species lacks a large tubercle on the dorsal surface of the acrocoracoid process. The femoral condyles appear to be stouter in the new species than the condition in *Lophogallus*^34–36^. The width of the third metatarsal trochlea in the new species is relatively stouter and shorter than that of *Diangallus*^33^. The new specimen has a third pedal digit relatively longer as compared to the tarsometatarsus, in contrast to the shorter condition in *Linquornis*^31^. It also differs from extinct species of *Syrmaticus* in the morphology of the minor metacarpal^34–36^, and from *Plioperdix* in having a larger intermetacarpal process^34–36^.

### Description and comparison

The new specimen represents a medium-sized species of phasianin similar in size to eared pheasants (*Crossoptilon*), with longer and robust hind limbs, but with relatively shorter forelimbs (see Table 1 for measurements). The specimen is exposed primarily in left lateral view (Fig. 1). The skull is poorly preserved and the premaxillae, nasals, maxillae, and bones of the frontal-orbital region are partially reconstructed with plaster (Fig. 1, highlighted in grey). The basicranium and jaw are original bony material. The elongated postorbital process fuses with the zygomatic process distally, a feature shared with other phasianids^27^. No foramen is present on the lateral surface of the caudal mandible, as opposed to a large foramen present on the mandible in guans (*Penelope*), turkeys (*Meleagris*), and grouse (Tetraoninae)^38^.

The cervical vertebrae are incompletely preserved, and the cranial part of the series is reconstructed with glue. Two caudal cervical vertebrae are preserved *in situ* and in articulation (Fig. 1). A lamina acrocostalis^38^ is present in these vertebrae. Neither a notarium, nor the morphology of the thoracic vertebrae are visible. Three articulated caudal vertebrae display long transverse processes and high spinal processes. The pygostyle is triangular in outline in lateral view, and its caudal tip extends only slightly dorsally, differing from that in turkeys (*Meleagris*) and guineafowl (*Guttera*), in which the distal pygostyle strongly projects dorsally. A weak ridge is discernible on the ventral side of the pygostyle in the new specimen (Fig. 2).

Several sternal and thoracic ribs are preserved, overlain by the left hind limb (Fig. 2). The thoracic ribs are mediolaterally thin but craniocaudally wide, with fused elongate and wide uncinate processes. The furcula is partially exposed and the rami appear to meet at the apophysis to form a ‘V-shape’ as in other phasianids, but this region is partially obscured by matrix. The omal ends of the furcula are expanded (Fig. 2). The omal end of the left coracoid is partially exposed in ventrolateral view. The acrocoracoid process is hooked medially, with a shallow impression marked the cranial surface (Fig. 2). The proximal right scapula is only partially visible in medial view, and the scapular acromion extends cranially. The coracoid tubercle is slightly convex.

The cranial margin of the sternum is slightly concave in outline, but less concave than the curvature in most phasianids (e.g., *Francolinus*). The carinal apex of the sternum is positioned caudally relative to the external sternal rostral spine as in crown galliforms^38^, and the apex also is caudal to the cranial edge of the sternal incisures as in *Lophophorus*, *Bonasa*, and species with an enlarged crop^38^. The sternal body is similar to turkey and grouse in the dorsoventral width of the keel, which is greater than most other phasianids. Two caudal incisures (the left and right incisura lateralis^39^) are visible (Fig. 2). The cranial extension of the lateral incisure is quite deep compared to that of *Crax* or *Acryllium*, and similar to other phasianids. The width of the lateral trabecular arm of the new specimen is similar to other pheasants, but narrower than that in cracids and megapodes. The medial trabecula is longer and narrower than the lateral, with a shallow groove on the external surface. The craniolateral process of the sternum is not preserved. The mediolateral thickness of the keel varies along its ventral edge (Fig. 2) and is thickest in its cranial portion. The coiled trachea abuts the cranial margin of the sternal keel (Fig. 2). There is no evidence of a crop being preserved in this region of the specimen.

The left humerus is visible in cranial view, and the right one only has part of the humeral head exposed (Fig. 2). The deltopectoral crest deflects dorsally, and a tubercle is present on the cranial surface of the crest (Fig. 3). The second (dorsal) pneumatic fossa (pneumotricipital fossa) on the proximal humerus appears to be shallow or absent. The dorsal condyle of the humerus is rounded and appears to be longer than the ventral condyle. The ulna is robust and is slightly shorter than the length of the humerus. Only the dorsal ramus of the ulnare is exposed, and a concave facet for articulation with the dorsal condyle of the ulna is visible. The left carpometacarpus is preserved in dorsal view. The intermetacarpal process is present with a triangular shape and does not fuse to the minor metacarpal (Fig. 3). However, it slightly overlaps the edge of this metacarpal. By contrast, the intermetacarpal process is only weakly developed in Odontophoridae^25^. The intermetacarpal process of *Panraogallus* is shorter than that in Tetraoninae and similar to *Tragopan* and other phasianids. The extensor sulcus is visible on the distal half of the major metacarpal (Fig. 3). The minor metacarpal is bowed caudally, less than that of Cracidae^25^, with its apex distal to the intermetacarpal process. The minor metacarpal bears a concave cranial surface with a flange protruded caudally (Fig. 3). The supratrochlear surface is decorated with tiny pits and bulges, unlike the larger tubercles present in *Lagopus*. As in many extant members of Phasianidae (e.g., *Alectoris*), a long and slim ossified tendon is present adjacent to the major metacarpal. The alular digit is displaced adjacent to the carpometacarpus (Fig. 3, ad-1). The extensor process of the carpometacarpus is stout (Fig. 3).

### Pelvis and hindlimb

The dorsoventral height of the ischium is proportionally similar to that of other phasianids, but much higher than that of Tetraoninae (e.g., *Dendragapus, Lagopus*, and *Tetrao*). The fenestra between the ischium and pubis is a narrow slit with the pubis closely aligned with the ventral edge of the ischium (Figs. 2 and 3). The terminal process of the ischium is missing and appears to have been pointed and straight based on the impression of the missing bone preserved in the matrix. A notch is present in the distal end of the ischium in grouse (Tetraoninae)^40^, but does not appear to have been present in the new specimen. The dorsolateral crests of the ilia are projected slightly. The antitrochanter rises into a small tubercle, but it is largely obscured (Fig. 2). The preacetabular tubercle (or pectineal process) appears to be small (Fig. 2) and shorter than that seen in *Phasianus*^25^.

The femur of the new specimen is slightly curved. The short and stout appearance of the femur is quite distinct from the much more slender and elongate femur of Cracidae and Megapodiidae. A femur longer than the humerus is in contrast to the state present in Cracidae, Numididae, Odontophoridae, and Tetraoninae^25^. The projection of the trochanteric crest of the left femur in the fossil is greater than that of Megapodiidae, but less than in Cracidae^27^. The four scars on the proximolateral surface of the femoral shaft are mostly similar to other members of Phasianinae (e.g., *Lophophorus*) (Fig. 4). In lateral view, the central scar is round and associated with the attachment of puboischiofemoralis muscle (PIFE in Fig. 4). A longer groove is cranially positioned and deeply marked, and it is associated with the attachment of the iliotrochantericus caudalis muscle (Fig. 4: ITC). A few smaller trough-like ridges associated with the ischiofemoralis and iliotrochantericus cranialis muscles (Fig. 4: ISF and ITCR) are positioned distal to the ITC (Fig. 4). The medial and lateral condyles are well projected as seen in the right femur, with the medial one wider than the lateral one; a deep groove (intercondylar incisure^39^) is present in between the condyles.

The right tibiotarsus is visible in cranial and medial views (Fig. 5). The femoral articular facet of the tibiotarsus is visible (Fig. 6), and the facet is slightly narrower mediolaterally than its craniocaudal length. A ball-shaped interarticular area bulges in the center of the facet^39^. Cranial to this bulge, the retropatellaris fossa is developed. Medial to the bulge, the medial articular facet is convex. The tibial incisure is significantly concave, and the patellar crest protrudes distinctly (Fig. 5). The cnemial crests do not extend far proximally relative to the proximal end of the tibiotarsus. The fibular crest projects laterally with a long intermuscular ridge extending distally. Distally, the medial and lateral condyles of the tibiotarsus are subcircular in outline. The ossified superatendinal bridge is present but broken. A narrow extensor sulcus is present on the distal end of the tibiotarsus. The distal medial condyle projects cranial to the lateral condyle. A crescent-shaped sesamoid is preserved positioned against the distal ends of the left and right tibiotarsi (Fig. 5).

The tarsometatarsi are long and are similar to the length of the femur (Fig. 5; see Table 1 for measurements). They are relatively stouter than those of the long-legged Megapodiidae and Numididae. Comparatively, the tarsometatarsus in the fossil is longer than that of most other phasianids (e.g., Tetraoninae). The left tarsometatarsus is visible in plantolateral view and the right, in medial view (Figs. 2 and 5). The plantar opening of the distal vascular foramen is rounded. The hypotarsal crests are short overall, but the medial hypotarsal crest extends distal to the lateral crest. A blunt tarsometatarsal callosity that potentially associated with further attachment of the spur is preserved on the left tarsometatarsus just distal to the proximodistal midpoint of the bone. A spur is present in males of several galliform taxa including Meleagridinae and Phasianinae, and it is absent in Odontophoridae, Numididae, and Tetraoninae^41^. The lateral cotyle of the tarsometatarsus is slightly deeper than the medial one. The medial parahypotarsal fossa is deep (Fig. 5).

**Table 1:** Major measurements of the holotype specimen (in mm).

Skeletal Elements	Left (mm)	Right (mm)	Middle shaft width left (mm)	Middle shaft width right (mm)	Individual tracheal ring diameter (mm)	Ring height (mm)
Humerus	74.0		7.2		**6.8**	**4.2**
Ulna	69.2		6.3		**5.4**	**3.6**
Radius	64.8		3.4		**6.0**	**3.5**
Carpometacarpus	38.3		3.3 (Major metacarpal)		**5.0**	**3.0**
Femur	81.5		6.8		**5.9**	**4.0**
Tarsometatarsus	76.0	75.2	7.5		**6.1**	**5.0**
Tibiotarsus	112.1	116.6	6.1	5.8	**5.0**	**3.4**
Fibula	61.9				**5.4**	
Post-acetabular portion	47.3				**5.0**	
Pelvic depth	23.8				**6.5**	
Pygostyle	16.0				**7.6**	
Sternal width	53.2				**7.3**	
Pedal Digits						
I-1	10.5	10.5				
II-1		18.1				
II-2		13.7				
III-1	17.8	19.3				
III-2	14.3	16.2				
III-3	11.2	12.4				
IV-1	11.5					
IV-2	7.3	7.9				
IV-3	5.9	6.7				
IV-4	7.8	8.6				
Ungual-1		7.9				
Ungual-2	8.5					
Ungual-3	8.4	10.3				
Ungual-4	8.7	8.7				

The metatarsal trochleae are not widely splayed (Fig. 2). Metatarsal trochlea II is plantarly deflected (relative to metatarsal trochlea III), and extends approximately as far distally as metatarsal trochlea IV. The trochlea of metatarsal II bears a plantarly projected wing (Fig. 5). Metatarsal trochlea III is almost symmetric in distal view. Metatarsal trochlea IV bears rounded lateral depression (fovea. lig. collat.^39^). The hallux is elevated proximally on the tarsometatarsal shaft, near the distal end of the medial plantar ridge along the tarsometatarsus shaft. The phalanges are well preserved with digit III being the longest, and despite its length, that digit is slightly shorter than the length of tarsometatarsus (Fig. 5). Generally, the pedal (non-ungual) phalanges decrease in length distally, and the ungual phalanges are shorter than the penultimate phalanx in length. The pedal ungual phalanges are somewhat curved with a lateral sulcus (Figs. 2 and 5). Phalangeal proportions indicate that this bird likely was terrestrial in its habits^42^.

Approximately 40 ossified tracheal rings are preserved around the pectoral region of the skeleton with some additional isolated rings in the abdominal region of the body. The exterior surface of the tracheal rings appears tuberculated in subparallel patches (Fig. 2, pat). The color of these mineralized tracheal rings is reddish-grey and is distinguished from the white color of other skeletal features (Fig. 2). This difference would be consistent with the comparatively weakly ossified or mineralized cartilage of tracheal rings in extant species and subsequent diagenetic changes. There does not appear to be a large variation in tracheal diameter over the length of preserved trachea, or any isolated rings (n = 12, mean = 6.0 mm, Stdv = 0.9). Given the missing tracheal rings that would have been anatomically closer to the head or within the body cavity (adjacent to the lungs), it is not clear if the trachea had a greater diameter in the elongated region, as in some extant species. If the more cranial portion of the trachea is present in the fossil, it likely would be located along the right side of the cervical vertebrae as in most crown birds and all known galliforms, when in life position^43,44^.

Cranial to the sternal margin and keel, approximately 25 tracheal rings are aligned to form coiled shapes. We believe that this coiling of the trachea is not an artifact of preservation because the uncoiled length of the trachea as preserved (minimum of ~36.2 cm) would extend beyond the position of the head, and clearly points to the presence of a hyper-elongated trachea. The particular coiling pattern preserved in the fossil (Fig. 2) is most similar to that seen in grouse^11^. The proximodistal width of individual tracheal rings ranges between 3–4 mm, significantly larger than that of most other Galliformes, but rather close to some large-sized Anseriformes with tracheal elongation, such as the ring diameters of *Anseranas semipalmata* ranged from 5–7 mm (American Museum of Natural History 1772, 1873, and 2771).

The coiled section of the trachea appears to form at least two complete loops with approximately half of another loop preserved. The great diameter of the individual tracheal rings in the coiled, elongated portion of the trachea is consistent with the increased diameter and greater tracheal ring width present in living birds with elongate tracheas^12,13^. The more caudal section of the trachea preserved on the sternal keel and the isolated tracheal rings in the abdominal region also display similar wide tracheal rings and a diameter consistent with the coiled section cranial to the sternal keel, rather than the smaller and incomplete tracheal rings that likely would be present adjacent to the lungs in extant species^13,43^. In addition, it would also be extremely difficult to have broken the trachea close the lungs and have it moved outside the thoracic cavity, then laterally and cranially to position them on the sternum (without disturbing other parts of the body/skeleton). Thus, the sternal keel section of the trachea could be a caudally displaced section that broke away from the main coiled part. However, that section might also be a representative of an elongate subcutaneous coil along the side of the body, and its position and orientation (in line with the long axis of the body) are consistent with that conclusion (Fig. 2). The additional isolated rings in the abdominal region suggest that such a loop could have extended further caudally as in some cracids and birds of paradise, where the trachea reaches the abdominal and rear areas of the body^13,14^. If that caudal section of trachea derived from a long subcutaneous loop, it would indicate that the trachea was at least ~13 cm longer than our conservative estimate based on the coiled region (maximum length over 49 cm). For comparison, the length of the trachea in chicken ranges from 15.5 to 18 cm^45^. It appears that the trachea was at a minimum longer than the length of the bird’s body (36 cm vs. 30 cm, an estimate of length from head to tail). Lastly, the coiling of the trachea and its close application to the cranial edge of the sternal keel (and cranial to the furcula) suggests that the crop and interclavicular air sacs would have been displaced or restricted in some way.

## Discussion

**Figure 7:**
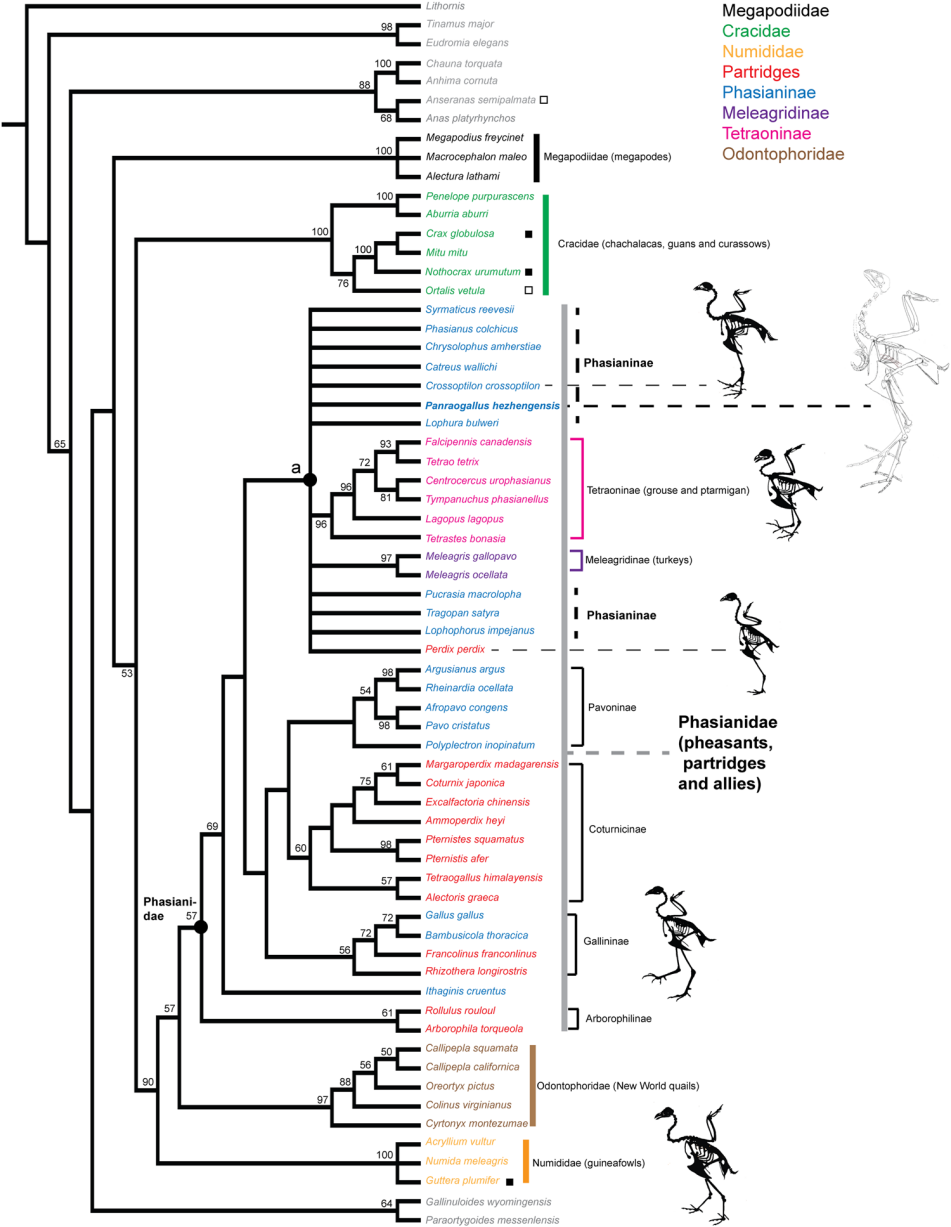
Strict consensus of the most parsimonious trees showing the phasianin affinity of *Panraogallus hezhengensis*. Major clades of galliforms are colored. Tracheal elongation is labeled on terminal branches with dots, and its distribution suggests multiple origins within Galloanserae (closed squares indicate that both sexes have the trait, and open squares designate that only males have the trait).

The phylogenetic analysis (see methods) resulted in 24 most parsimonious trees (with a length of 11792 steps) in PAUP, and the strict consensus of those trees places the new species within a polytomy comprised of species of Phasianinae, Tetraoninae, and Meleagridinae (node ‘a’ in Fig. 7). Other major clades of crown Galliformes (Megapodiidae, Cracidae, Numididae, Tetraoninae, and Odontophoridae) were recovered as monophyletic, and their relationships largely are consistent with results of prior analyses using either morphological or molecular datasets, or both^28,46^. Within Phasianidae, the clades Tetraoninae, Pavoninae, Coturnicinae, and Gallininae are all well supported (see bootstrap support in Fig. 7).

The new specimen is referred to crown Galliformes by the following characters: two caudal incisures present on the sternum^27,28^; cranial apex of the sternal keel shifted caudally; the rami of the furcula narrow; and a tarsometatarsus with two hypotarsal crests, between which a long groove is present^27,28^. The specimen is supported as part of Phasianidae by the presence of following synapomorphies: a relatively wide intermetacarpal space; metatarsal trochleae II and IV closely spaced and subequal in distal extent; hallux proximally situated on the tarsometatarsus; and four distinct muscular scars are present on the lateral surface of the proximal femur^26^. This specimen displays a tarsometatarsal process for spur attachment that is only known to be present in Numididae, Phasianinae, and Meleagridinae^30^. The new specimen can additionally be differentiated from Tetraoninae by the absence of a dorsoventrally narrow ischium^40^ and from Odontophoridae by the arrangement of proximolateral muscular scars on the femur^26^. Both of these taxa also lack the tarsometatarsal spur^37^. Finally, the length of the humerus is less than that of the femur (Figs 2 and 3; see Table 1), a synapomorphy of crown Phasianinae^38,47^. The holotype specimen of *Panraogallus* is distinguished from other galliform species not only by discrete characters, but also by size and limb proportions.

Mineralized or ossified cartilaginous tracheal rings are preserved and reveal a highly elongated trachea that is rarely present in phasianids. This trait has evolved independently at least four other times among galliform species, including in grouse (e.g., *Tetrao urogallus* and *Lagopus mutus*), cracids (e.g., *Ortalis vetula*), and guineafowl (e.g., *Guttera plumifera*)^11,32^. The reconstructed coiling pattern of *Panraogallus* is more similar to *Tetrao urogallus* in forming a ‘zigzag’ shape, rather than to other simpler looped coiling patterns present in cracids and guineafowl^32,48^ (Fig. 2). The tracheal coiling preserved here represents the earliest fossil evidence of this acoustically important trait. The point-like projections on the exterior surface of the tracheal rings have not been described in extant species (and may be an autapomorphy of this extinct species). These pointed structures in the fossil trachea might serve to increase the attachment between cartilaginous rings and the surrounding epithelium. The close contact between cartilage and surrounding tissues provides better heat exchange function than a simple, smooth surface.

The independent evolution of tracheal modification in pheasants is consistent with the broader convergent pattern among galliforms, suggesting possible similar underlying factors in selection. We further examined five traits in comparative analyses (using ‘R,’ see Electronic supplementary material), including TE, esophageal inflation, mating system, and habitat openness. However, our results demonstrate that none of those characteristics exhibit any significant correlation to one another on the most parsimonious trees in our analyses (Methods and Supplementary Figs 1 and 2). Therefore, the evolution of TE in galliforms does not appear to have been directly influenced by those factors. The body mass of the new species is estimated to be approximately 2.5 kg based on regression of femoral length^49^. That mass estimate is much greater than the average mass of galliform species without a TE (~0.83 kg) and similar to the mean value of galliform taxa with a TE (~2.26 kg^11^). Previous work did not treat body mass and TE in a comparative framework^11^. Here we show that evolutionary origins of TE are linked to increases in body size (see Electronic supplementary material). More anatomical work may identify further cases of TE in birds, but our results are robust to additional gains of TE (Supplementary Fig. 3).

*Panraogallus* likely produced a lower frequency vocalization and/or louder call than its close phasianine relatives (of similar size) implied by its elongate, coiled trachea and larger body size. Such lower frequencies likely occurred later in ontogeny during what has been termed ‘voice breaking,’ and that change is not necessarily directly linked to reaching a certain size or ontogenetic stage because TE (growth) is complete before the change in vocalization pattern^50^. *Panraogallus* represents an independent evolution of an elongate trachea and the third record of such elongation in the diverse clade Phasianidae (Fig. 7). Its independent evolution in a savannah/open habitat setting may be consistent with the greater frequency bandwidth produced by some birds in open habitats relative to their body size and phylogenetic constraints^17,51^. The elongate trachea^52^ most often co-occurs with larger body sizes (Supplementary Figs 4 and 5) that are associated with lower frequencies and potentially louder calls^11^. Given the preference of the ‘size exaggeration’ hypothesis for the evolution of the TE in birds^11^, and that *Panraogallus* has a body mass consistent with other galliforms with elongate tracheae, it is possible (if not likely) that this extinct Chinese species produced calls of a lower fundamental frequency and possibly louder calls than its similar sized close relatives. Potential selective advantages of low frequency calls include territory defense and mate attraction^52,53^. Such vocalizations should have carried a long distance in the reconstructed temperate savannah habitat where *Panraogallus* lived in the Linxia Basin^23,24^. While the data are consistent with the ‘size exaggeration’ hypothesis, we cannot reject other hypotheses and functions for tracheal elongation in this extinct species such as it being a compensative mechanism for heat conservation and water retention in the late Miocene^54^.

The co-occurrence of a bony process for attachment of the spur on the tarsometatarsus and an elongate trachea, features frequently present in males of sexually dimorphic species of galliforms and other birds^13,15,41,55,56^ may indicate that the holotype specimen is of a male individual. The evolutionary gain of vocal modification (and potential vocal dimorphism) in an extinct lineage of pheasants is more consistent with the tracheal modification acting as a competitive trait (more frequently gained by males) rather than a signaling trait (e.g., plumage color) that are lost more commonly in phasianid evolution^55^. The presence and degree of sexual dimorphism among phasianids is evolutionarily labile^41^ and the discovery of *Panraogallus* demonstrates that the evolutionary history of pheasants is more complex than previously hypothesized.

## Conclusions

The nearly complete skeleton of *Panraogallus hezhengensis* includes an elongate, coiled trachea that also appears to have possibly extended subcutaneously over the pectoral muscles to the posterior abdominal region. The preserved tracheal rings are wider and thicker than typical galliform rings but consistent with the morphology present in other bird species with tracheal elongation^13^. The unexpected preservation of the coiled elongate trachea in the new species likely indicates an acoustic adaptation independently evolved within an extinct galliform lineage, and that this type of vocal modification likely was more widespread across birds in the past. The co-occurrence of an elongate trachea and a tarsometatarsal projection for the attachment of the spur in this individual might represent sexually dimorphic features only present in males. Given the diversity of birds that have evolved TE and the absence of correlation of its evolution with many other biological and environmental factors, future work should examine the genetic and developmental modifications that lead to the formation of TE in order to determine the underlying mechanism for its production. Such data likely will shed more light on the reason(s) why this vocal modification has repeatedly evolved among crown group birds.

## Methods

### Phylogenetic analysis

In order to test the phylogenetic position of the holotype specimen of *Panraogallus hezhengenesis*, we analyzed a combined molecular and morphological character matrix^28^ in PAUP^57^ containing 120 morphological characters (with six characters ordered, 35–37, 55, 67, and 109) and 5022 BP of molecular sequences (Supplementary data). Paleognaths and anseriforms were used as the outgroups to the galliform taxa. A heuristic search was done using 3000 random taxon addition sequences and tree bisection and reconnection branch swapping. Zero length branches were collapsed. 1000 replicates of bootstrap were conducted in PAUP, and support numbers larger than 50% are reported.

### Comparative analyses

We reconstructed joint ancestral states for four life history and morphological characters using squared-change parsimony since branch lengths are not in units of time. Branch lengths were transformed to length 1 using compute.brlen() in the R package ape^58^. The four traits used in analyses are: tracheal elongation, esophageal inflation, mating system, and habitat openness (see Supplementary Methods for details). We used a maximum likelihood approach in the corDISC() function of the R package corHMM^59^ because it allowed us to test for trait correlations with missing data (coded as ‘0&1’) and to incorporate polytomies. We tested for correlated evolution using a modified Pagel’s test^60^. Briefly, we fit two models: one in which all rates between different trait combinations were allowed to vary independently and one in which trait evolution was not affected by the state of the other traits^60^. We then selected the best model used AICc, an adjusted version of Akaike information criterion (AIC) suitable for small sample sizes^61^. We ran this analysis over all 24 parsimony trees and averaged the results.

To test for a correlation between body size (ln mass in grams) and tracheal elongation (presence or absence), we used phylogenetic logistic regression in the phylolm package^62^. We used the phylogeny of ref.^5^ and data from ref.^11^. This analysis indicates that tracheal elongation is significantly correlated with the evolutionary increase in body size (beta = 0.49, p = 0.04, 100 bootstrap replicates). Given the limited amount of morphological data confirming presence or absence of TE in birds^11^, we further assessed the influence of missing data on our body mass analyses (see Supplementary Information).

### Data availability

The list of species and dataset is available in the supplemental dataset.

## Electronic supplementary material

SUPPLEMENTARY INFO

Supplementary Dataset
