# Supplementary material for: Vocal specialization through tracheal elongation in an extinct Miocene pheasant from China
Source: Sci Rep. 2018 May 25;8:8099. doi: 10.1038/s41598-018-26178-x (PMC5970207; doi:10.1038/s41598-018-26178-x)
Supplement: Supplementary file 1 — SUPPLEMENTARY INFO [file 41598_2018_26178_MOESM1_ESM.docx]

**Supplementary Information to:**

**Vocal specialization through tracheal elongation in an extinct Miocene pheasant from China**

Zhiheng Li^12^, Julia A. Clarke^3^, Chad M. Eliason^4^, Thomas A. Stidham^12^, Tao Deng^12^, and Zhonghe Zhou^12^

1 Key Laboratory of Vertebrate Evolution and Human Origins, Institute of Vertebrate Paleontology and Paleoanthropology, Chinese Academy of Sciences, Beijing, 100044, China

2 CAS Center for Excellence in Life and Paleoenvironment, Beijing, 100044, China

3 University of Texas at Austin, Department of Geological Sciences, Austin, TX 78712

4 Field Museum of Natural History, Integrative Research Center, Chicago, IL 60605

# **Supplementary methods and results**

## **Reconstruction ancestral states of tracheal elongation(TE) in pheasants**

Because branch lengths for obtained parsimony trees are not in units of time, we reconstructed ancestral states of five life history and morphological characters using squared-change parsimony. Branch lengths were transformed to length 1 using the compute.brlen() function in the ape R package. We looked at the relationship between tracheal elongation (TE) and three traits potentially linked to TE: 1) esophageal inflation (a potential adaptation for producing low-frequency sounds that would be predicted to be linked with TE under the redundant-signal hypothesis; see Kuijper et al. 2012), 2) mating system (with the prediction that TE should evolve more frequently in males of polygynous taxa because of a presumed greater strength of sexual selection; e.g., see Payne 1984), and 3) habitat openness (with the prediction that TE should evolve more frequently in closed habitats if TE allows birds to produce lower frequency sounds that can better propagate through dense habitats; see Fitch 1999). Habitat openness data was obtained from Del Hoyo et al., (1992), esophageal inflation data was obtained from Fitch (1999) and Riede et al. (2016), and mating system and TE data were obtained from Ksepka (2009) and Fitch (1999).

In all cases, the best-fitting model was one in which characters were allowed to evolve independently (i.e., no pairs of characters showed significant correlation). The ancestral state of tracheal length in Galliformes is estimated as non-elongate (Supplementary Fig. S1). Tracheal elongation then evolved independently at least 4 times in Galliformes (Fig. 7). Galliformes are estimated to have evolved in a closed habitat, with several subsequent transitions between open and closed habitats. Habitat openness was indeed more evolutionary labile (rates: q_gain_ = 0.25 ± 0.01, q_loss_ = 0.19 ± 0.01; values averaged across all trees) than tracheal elongation (q_gain_ = 0.04 ± 0.002, q_loss_ = 0.05 ± 0.12; values averaged across all trees).

## **Evaluating the relationship between body mass and tracheal elongation across birds**

The relationship between body mass and tracheal elongation (TE) was treated by Fitch (1999) but not in a comparative framework. A strong relationship between body mass and TE could arise simply from pseudoreplication (e.g., ~ 5000 passerine species presumed to lack TE). We therefore took a simulation approach to assess the sensitivity of our analysis to unknown or missing data. Briefly, we used the time-calibrated phylogeny of Riede et al. (2016), modified from Burleigh et al. (2015). This tree had 6712 species. We obtained body mass data for from Dunning (2007). After pruning tips from the tree without body mass data, we ended up with a tree with 5446 tips. Next, we obtained data for positive evidence of TE in birds from Fitch (1999). This resulted in 45 species identified as having TE. Species without data could either truly lack TE or be unknown (e.g., due to a lack of anatomical data). To address this uncertainty, we first assigned all species of the same genus in a monophyletic clade with all species in the same scoring class the same. This resulted in a modified dataset with 55 TE species. Next, we assessed sensitivity to missing trait data by assigning all species without evidence for TE as 1) non-TE in one dataset, 2) TE in another dataset, and 3) in various proportions of TE/non-TE through simulating trait data 5000 times with the rTraitDisc() function in the R package ape (Paradis 2004). Simulations were based on the rate estimated from the non-TE dataset under an equal rates model using fitDiscrete in the geiger package (Harmon et al. 2008). For each simulated dataset, we then ran phylogenetic logistic regression models using the phyloglm function in R (Ho and Ané 2014). R code and datasets used in these analyses is available on Dryad (DOI pending).

Assigning all species without data as non-TE showed a significant positive relationship between body size and TE (β = 0.81, p = 0.0032). Assigning all species with missing data as TE produced a non-significant, negative relationship between body size and TE (β = -0.084, p = 0.20). Sensitivity analyses based on simulated trait data (see Fig. S4) showed that the strength of the relationship between body mass and the number of species assigned as TE decreased strongly at around 70 species (Fig. S5). This suggests that our result of a significant relationship between body mass and TE for all unknown species assigned as non-TE is robust up to ~ 25 new species that may be found in the future to have tracheal elongation.

# **Supplemental References**

Beaulieu, J. M., O'Meara, B. C., & Donoghue, M. J. (2013). Identifying Hidden Rate Changes in the Evolution of a Binary Morphological Character: The Evolution of Plant Habit in Campanulid Angiosperms. *Systematic Biology*, *62*(5), 725–737. http://doi.org/10.1093/sysbio/syt034

Burleigh, J. G., Kimball, R. T., & Braun, E. L. (2015). Molecular Phylogenetics and Evolution. *Molecular Phylogenetics and Evolution*, *84*(C), 53–63. http://doi.org/10.1016/j.ympev.2014.12.003

Del Hoyo, J., Elliot, A. and Sargatal, J., 1992. Handbook of the Birds of the World. Barcelona: Lynx Editions.

Dunning, J. B. (2007). CRC Handbook of Avian Body Masses, Second Edition. Boca Raton, FL: CRC Press.

Fitch, T. (1999). Acoustic exaggeration of size in birds via tracheal elongation: comparative and theoretical analyses. *Journal of Zoology*, *248*(1), 31–48.

Harmon, L. J., Weir, J. T., Brock, C. D., Glor, R. E., & Challenger, W. (2008). GEIGER: investigating evolutionary radiations. *Bioinformatics*, *24*(1), 129–131. http://doi.org/10.1093/bioinformatics/btm538

Ksepka, D. T. Broken gears in the avian molecular clock: new phylogenetic analyses support stem galliform status for *Gallinuloides wyomingensis* and rallid affinities for *Amitabha urbsinterdictensis*. *Cladistics*, **25**, 173–197, (2009).

Kuijper, B., Pen, I., & Weissing, F. J. (2012). A Guide to Sexual Selection Theory. *Annual Review of Ecology Evolution and Systematics*, *43*(1), 287–311. http://doi.org/10.1146/annurev-ecolsys-110411-160245

Pagel, M. (1994). Detecting correlated evolution on phylogenies - a general-method for the comparative-analysis of discrete characters. *Proceedings of the Royal Society of London Series B-Biological Sciences*, *255*(1342), 37–45.

Paradis, E., Claude, J., & Strimmer, K. (2004). APE: analyses of phylogenetics and evolution in R language. *Bioinformatics*, *20*(2), 289–290.

Payne, R. B. (1984). Sexual selection, lek and arena behavior, and sexual size dimorphism in birds. *Ornithological Monographs*, *33*, 1–52.

Riede, T., Eliason, C. M., Miller, E. H., Goller, F., & Clarke, J. A. (2016). Coos, booms, and hoots: The evolution of closed-mouth vocal behavior in birds. *Evolution*, *70*(8), 1734–1746. http://doi.org/10.1111/evo.12988

Tung Ho, L. S., & Ané, C. (2014). A Linear-Time Algorithm for Gaussian and Non-Gaussian Trait Evolution Models. *Systematic Biology*, *63*(3), 397–408. <http://doi.org/10.1093/sysbio/syu005>

# **Supplemental Figures**

**Figure S1. Ancestral states of tracheal elongation and habitat openness.** Squares at tips of tree show data for habitat openness (left square; open: white, closed: blue) and tracheal elongations (right square; white: TE absent, yellow: TE present). Gray squares indicate missing data. Pies at nodes show probability of ancestor being found in open habitats without TE (white), open habitats without TE (blue), open habitats with TE (yellow), and closed habitats with TE (green). For example, the ancestor of *Ortalis vetula*, *Mitu mitu*, and *Crax globulosa* is inferred as having an elongated trachea and living in closed habitats. Ancestral states were reconstructed using squared changed parsimony, accounting for uncertainty in trait scorings and polytomies.****

**Figure S2. Ancestral states of tracheal elongation (TE) and esophageal inflation (EI).** Squares at tips of tree show data for esophageal inflation (left square; EI absent: white, EI present: blue) and tracheal elongation (right square; white: TE absent, yellow: TE present). Gray squares indicate missing data. Pies at nodes show probability of ancestor having either EI (blue) or TE (yellow), or both (green). Ancestral states were reconstructed using squared changed parsimony, accounting for uncertainty in trait scorings and polytomies.

**Figure S3. Ancestral states of tracheal elongation and mating system.** Squares at tips of tree show data for mating system (left square; monogamous: white, polygynous: blue) and tracheal elongations (right square; white: TE absent, yellow: TE present). Gray squares indicate missing data. Pies at nodes show probability of ancestor having either TE (yellow) or a polygynous mating system (blue), or both (green). Ancestral states were reconstructed using squared changed parsimony, accounting for uncertainty in trait scorings and polytomies.

**Figure S4. Method for addressing tracheal elongation (TE) in species lacking anatomical data.** Phylogeny for all birds showing species known to have TE (red), along with a simulated dataset for species with unknown tracheal anatomy (blue). Trait data was simulated in this way 5000 times and analyses were then conducted on each simulated dataset to understand the influence of missing data on the inferred relationship between body mass and TE (see Fig. S5). Phylogeny taken from Burleigh et al. (2015) with branch lengths from Riede et al. (2016).


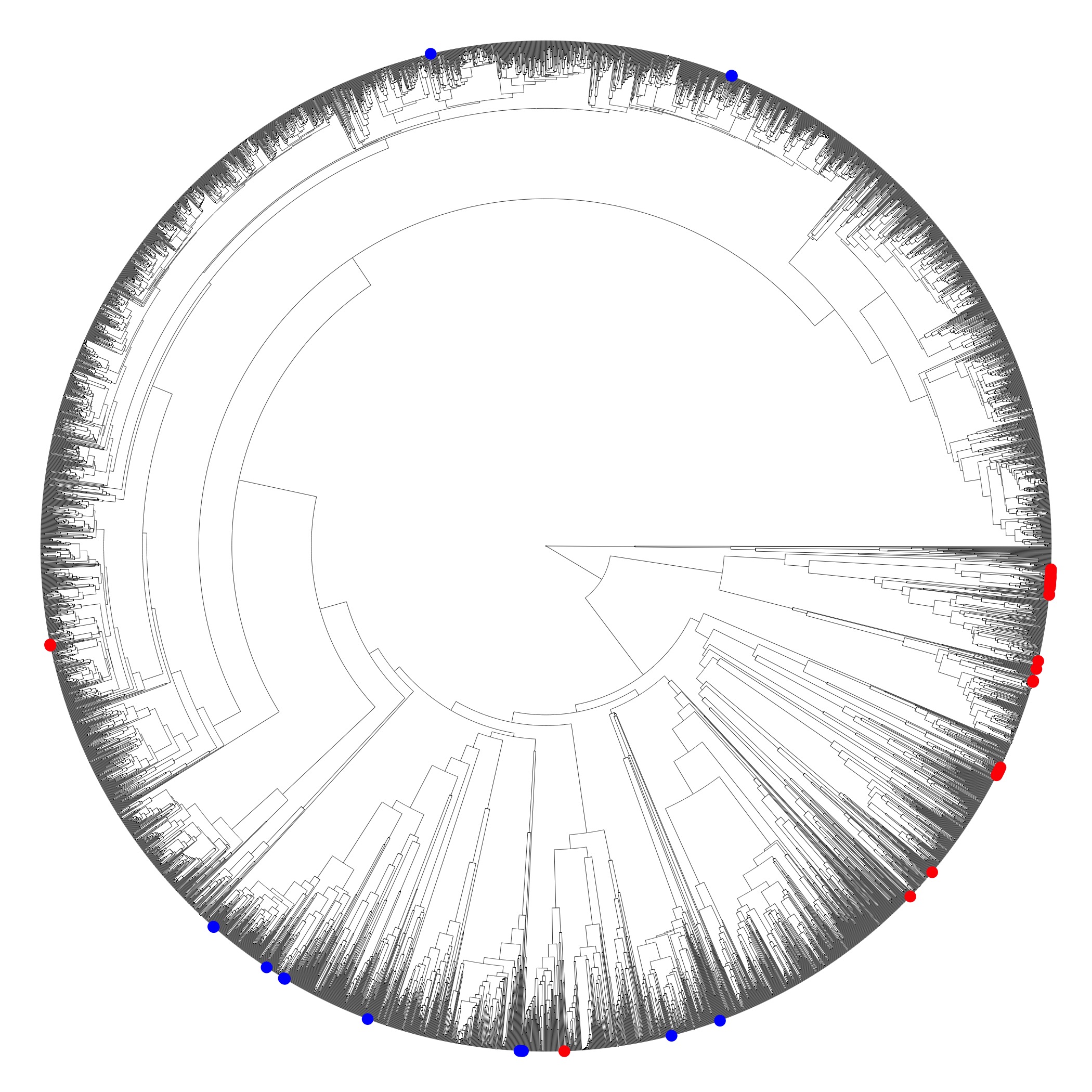


**Figure S5. Assessing the effect of missing tracheal elongation (TE) data on the relationship between body mass and tracheal elongation in birds.** Points show strength and direction of the relationship between body mass and TE for different numbers of species lacking data assigned as having TE (see Fig. S4). Each point represents a single model fit based on one of 5000 simulated datasets using the phylogeny of Burleigh et al. (2015) with branch lengths from Riede et al. (2016). Color of points indicates significance of the relationship (i.e. p-values), with darker colors being more highly significant. Blue line is a loess smoothing function fit to the data. Dashed vertical line shows observed number of species (n = 45) with positive evidence for TE. Note that the x-axis has been log-transformed for ease of interpretation. The plot shows a strong drop-off for the effect size for the TE-body mass relationship at ~70 species. This suggests that if 25 point origins of TE are discovered across birds (e.g., with new dissection or CT scan data), the relationship between body mass and TE will become non-significant.
